# Development of a Novel Reverse Transcription Loop‐Mediated Isothermal Amplification Assay for the Rapid Detection of African Horse Sickness Virus

**DOI:** 10.1111/tbed.12549

**Published:** 2016-08-02

**Authors:** V. L. Fowler, E. L. A. Howson, J. Flannery, M. Romito, A. Lubisi, M. Agüero, P. Mertens, C. A. Batten, H. R. Warren, J. Castillo‐Olivares

**Affiliations:** ^1^ The Pirbright Institute Pirbright Woking Surrey UK; ^2^ ARC‐Onderstepoort Veterinary Institute Onderstepoort South Africa; ^3^ Laboratorio Central de Veterinaria‐Sanidad Animal Algete (Madrid) Spain; ^4^ OptiGene Limited Horsham West Sussex UK

**Keywords:** RT‐LAMP, African horse sickness virus, rapid detection, pen side, diagnostics

## Abstract

African horse sickness (AHS) is a disease of equids caused by African Horse Sickness Virus (AHSV) and is transmitted by *Culicoides* midges. AHS is endemic in sub‐Saharan Africa, but during the past century, outbreaks of significant economic importance and elevated mortality have been recorded in Northern African countries, the Iberian and Arabian Peninsula, the Middle East and the Indian subcontinent. Effective control combines the application of early warning systems, accurate laboratory diagnosis and reporting, animal movement restrictions, suitable vaccination and surveillance programs, and the coordination of all these measures by efficient veterinary services. Conventional reverse‐transcriptase (RT) PCR (RT‐PCR) and real‐time RT‐PCR (rRT‐PCR) assays have improved the sensitivity and rapidity of diagnosing AHS, resulting in the adoption of these methods as recommended tests by the World Organisation for Animal Health (OIE). However, currently these assays are only performed within laboratory settings; therefore, the development of field diagnostics for AHS would improve the fast implementation of control policies.

Loop‐mediated isothermal amplification (LAMP) is an isothermal, autocycling, strand‐displacement nucleic acid amplification technique which can be performed in the field. LAMP assays are attractive molecular assays because they are simple to use, rapid, portable and have sensitivity and specificity within the range of rRT‐PCR. This study describes the development of a novel RT‐LAMP assay for the detection of AHSV. The AHSV RT‐LAMP assay has an analytical sensitivity of 96.1% when considering an rRT‐PCR cut‐off value of *C*_T_ > 36, or 91.3% when no rRT‐PCR cut‐off is applied. Diagnostic sensitivity and specificity were 100%. This assay provides for a rapid and low cost AHS diagnostic for use in the field.

## Introduction

African horse sickness (AHS) is a viral disease of equids transmitted by biting midges of the genus *Culicoides*. African horse sickness is endemic to sub‐Saharan Africa; however, important outbreaks have occurred in North Africa, the Iberian Peninsula, the Middle East and the Indian subcontinent (Coetzer and Guthrie, 2004).

African horse sickness virus (AHSV) is a member of the genus *Orbivirus* within the family *Reoviridae* and is closely related to the virus causing bluetongue in ruminants. African horse sickness virus has a 10‐segmented genome of double‐stranded RNA contained within a non‐enveloped triple‐layer capsid. The virion is non‐enveloped and has an approximate size of 55–70 nm (Roy and Sutton, 1998). The outer capsid is formed by two major structural proteins, VP2 and VP5 (encoded by segments 2 and 6, respectively) that are involved in cell attachment and entry. VP2 is the most variable antigen of AHSV, determines the virus serotype, and is the main target of virus neutralizing antibodies (Burrage et al., 1993). The outer capsid sits on the outer‐core layer, formed by VP7 (encoded by segment 8), which is a highly hydrophobic, antigenically conserved and immuno‐dominant protein and is the basis of ELISA‐based diagnostic tests (Chuma et al., 1992; Laviada et al., 1992; Bremer et al., 1994; House et al., 1996 and Maree and Paweska, 2005). Likewise, the VP7 gene is most often used as the molecular target of reverse‐transcription polymerase chain reaction (RT‐PCR) and real‐time RT‐PCR (rRT‐PCR) (Zientara et al., 1993, 1995; Sailleau et al., 1997; Agüero et al., 2008; Fernandez‐Pinero et al., 2009; Quan et al., 2010; Guthrie et al., 2013). The inner core is formed by the highly conserved protein VP3, enclosing VP1, VP4 and VP6 proteins, (encoded by segments 1, 4 and 5, respectively) comprising the viral replicase complex, and the viral RNA genome segments. The four non‐structural proteins (NS1, NS2, NS3/3A and NS4) involved in virus assembly and morphogenesis, egress from the cell and control of innate immune responses are encoded by segment 6, segment 9 and segment 10 (Roy and Sutton, 1998; van Staden et al., 1998; Belhouchet et al., 2011; Manole et al., 2012).

African horse sickness is highly lethal in horses and usually only mild in donkeys, with mortality rates between 50 and 90% in susceptible populations (Mellor and Hamblin, 2004), while infection in zebras is usually asymptomatic (Coetzer and Guthrie, 2004). African horse sickness infection in horses usually causes an acute or hyperacute syndrome characterized by fever and the presentation of clinical signs associated with severe compromise of respiratory or cardio‐circulatory functions. These include, inappetence, depression, coughing, expectoration of transudate or blood from the respiratory tract with severe respiratory distress associated with lung oedema, as well as oedema of the head, eyelids, *supra*‐orbital fossae and neck and severe conjunctivitis. The febrile form is usually seen in horses with some degree of immunity and is associated with mild symptoms only, such as fever, congested conjunctivae and inappetence (Coetzer and Guthrie, 2004). The potential for rapid spread of AHS in immunologically naïve populations when the competent vector is present requires the need for rapid diagnostic techniques to inform control strategies.

Laboratory confirmation of clinical suspicion is made by the detection of AHSV in blood or post‐mortem tissues using classical virus isolation (VI), immuno‐histochemistry or antigen detection ELISA techniques (based on protein VP7) (Chuma et al., 1992; Laviada et al., 1992; Bremer et al., 1994; House et al., 1996 and Maree and Paweska, 2005). However, these methods have now been superseded by the adoption of more rapid, sensitive and accurate molecular diagnostic techniques such as RT‐PCR and rRT‐PCR (Zientara et al., 1993, 1995; Sailleau et al., 1997; Agüero et al., 2008; Fernandez‐Pinero et al., 2009; Quan et al., 2010; Guthrie et al., 2013). These assays are widely used in most AHSV diagnostic laboratories and are the recommended tests by the OIE (World Organisation for Animal Health) (OIE, 2015) not only for confirmation of clinical diagnosis but also for animal certification purposes, import/export testing and surveillance.

While current rRT‐PCR methods are accurate, rapid and sensitive, they are required to be performed in laboratory settings and samples must be transported under the appropriate conditions from the point of collection to the laboratory. This process can delay the confirmation of suspicion of AHSV infection and the implementation of adequate control measures. Previous outbreaks of AHS in Europe demonstrated that these delays favoured the spread of the disease in the region and complicated its eradication (Portas et al., 1999; Sanchez‐Vizcaino, 2014). The development of rapid field diagnostic assays that can be used at the point of sample collection would enable the fast implementation of animal movement controls and would improve the control of a potential outbreak.

Loop‐mediated isothermal amplification (LAMP) is an isothermal, autocycling, strand‐displacement DNA amplification technique (Notomi et al., 2000) which can combine a reverse‐transcription step (RT‐LAMP), be performed at a single temperature in a water bath and can be combined with simple, disposable visualization using molecular lateral‐flow devices (LFD's) (Waters et al., 2014; Howson et al., 2017; Fowler et al., 2016). Numerous LAMP assays have been developed for both human and veterinary pathogens including orbiviruses such as bluetongue (Mulholland et al., 2014; Mohandas et al., 2015; Maan et al., 2016), however, as yet there has not been one developed for detection of AHSV. This study describes the development and performance of a novel RT‐LAMP assay for detection of AHSV, benchmarked against one of the OIE recommended rRT‐PCR assays (Agüero et al., 2008).

## Material and Methods

### Virus isolates and clinical specimens

Diagnostic sensitivity and specificity of the RT‐LAMP assay were evaluated using two panels of samples prepared at Laboratorio Central de Veterinaria‐Sanidad Animal (Madrid, Spain) for an AHSV International Ring Trial (Table 1). Panel 1 comprised 50 horse field samples submitted to Onderstepoort Veterinary Institute (OVI), South Africa, between December 2013 and May 2014. At the laboratory of origin (OVI), 44 samples tested positive and six tested negative by the RT‐PCR method described by Bremer et al. (1994). Panel 2 comprised 45 negative horse blood samples each artificially spiked with different concentrations of inactivated AHSV strains. African horse sickness virus strains were used at a dilution of 10^−4^ in AHSV‐negative horse blood to produce an expected *C*_T_ value of 30 by the gold standard rRT‐PCR test (Agüero et al., 2008). In addition, the panel included 10‐fold dilution series (10^−2^–10^−7^) of the Sen2007 and Eth2008 AHSV strains to determine the analytical sensitivity of the assay and bluetongue virus (BTV‐8) and equine encephalosis virus (EEV) for specificity testing.

**Table 1:** Virus isolates and clinical specimens

Sample ID	Sample details^a^	rRT‐PCR mean *C*_T_	RT‐LAMP mean T_p_	RT‐LAMP mean *T*_a_
A1/16 25	Field blood; ++	43.05	No *T*_P_	No *T*_a_
A1/16 26	Field blood; +++	34.34	16.13	87.89
A1/16 27	Field blood; +++	31.52	25.84	87.58^b^
A1/16 28	Field blood; ++	28.67	12.64	87.76
A1/16 29	Field blood; ++	25.45	12.9	87.59
A1/16 30	Field blood; ++	29.83	13.36	87.79
A1/16 31	Field blood; +++	26.23	11.64	87.79
A1/16 32	Field blood; +	Undet.	No *T*_P_	No *T*_a_
A1/16 33	Field blood; ++	36.87	16.33	87.81
A1/16 34	Field blood; ++	32.53	17.92	87.50^b^
A1/16 35	Field tissue; +++	29.69	13.04	87.37
A1/16 36	Field blood; ++	38.49	14.95	87.71
A1/16 37	Field blood; +++	43.61	No *T*_P_	No *T*_a_
A1/16 38	Field blood; –	Undet.	No *T*_P_	No *T*_a_
A1/16 39	Field blood; +++	29.43	16.28	87.22
A1/16 40	Field blood; +++	27.62	18.37	87.39
A1/16 41	Field blood; +++	25.05	11.66	87.63
A1/16 42	Field blood; +++	28.17	29.03	87.63
A1/16 43	Field blood; +++	29.59	12.53	87.51
A1/16 44	Field tissue; ++	34.92	No *T*_P_	No *T*_a_
A1/16 45	Field blood; +	28.08	21.71	87.51
A1/16 46	Field blood; +	32.04	13.37	87.31
A1/16 47	Field blood; +	29.11	14.18	87.77
A1/16 48	Field blood; +	29.94	13.69	87.89
A1/16 49	Field blood; ++	27.40	20.9	87.54
A1/16 50	Field blood; +++	30.60	15.52	87.59
A1/16 51	Field blood; +	29.30	13.45	87.52
A1/16 52	Field blood; +	32.89	21.32	87.47
A1/16 53	Field blood; +	29.71	15.60	87.40
A1/16 54	Field blood; +	29.42	40.40	87.38
A1/16 55	Field tissue; +++	41.81	No *T*_P_	No *T*_a_
A1/16 56	Field blood; ++	26.93	11.99	87.89
A1/16 57	Field blood; ++	28.53	12.71	87.83
A1/16 58	Field blood; +	33.33	18.47	87.37
A1/16 59	Field blood; ++	32.56	12.63	87.79
A1/16 60	Field blood; ++	22.55	11.08	87.15
A1/16 61	Field blood; ++	29.36	13.53	87.42
A1/16 62	Field tissue; +	Undet.	No *T*_P_	No *T*_a_
A1/16 63	Field blood; ++	31.20	15.00	87.79
A1/16 64	Field blood; ++	27.23	20.36	87.69
A1/16 65	Field blood; ++	27.73	13.02	87.70
A1/16 66	Field blood; +	Undet.	No *T*_P_	No *T*_a_
A1/16 67	Field blood; ++	29.49	11.45	87.62
A1/16 68	Field blood; +	29.92	12.94	87.76
A1/16 69	Field blood; –	Undet.	No *T*_P_	No *T*_a_
A1/16 70	Field blood; –	Undet.	No *T*_P_	No *T*_a_
A1/16 71	Field blood; –	Undet.	No *T*_P_	No *T*_a_
A1/16 72	Field blood; –	Undet.	No *T*_P_	No *T*_a_
A1/16 73	Field blood; –	Undet.	No *T*_P_	No *T*_a_
A1/16 74	Field tissue; ++	27.07	12.96	87.65
A1/16 75	S.B.; N.D.; AHSV‐1; HS 29/62c; Nelspruit, South Africa; 1962	30.23	14.45	88.07
A1/16 76	S.B.; N.D.; AHSV‐2; HS 82/61c; South Africa; 1961	33.54	17.25	88.02
A1/16 77	S.B.; N.D.; AHSV‐3; HS 13/63; Malmesbury, South Africa; 1963	31.30	14.39	88.07
A1/16 78	S.B.; N.D.; AHSV‐4; HS 32/62; Zimbabwe; 1962	30.25	13.69	87.88
A1/16 79	S.B.; N.D.; AHSV‐5; HS 30/62c; South Africa; 1962	32.24	20.67	87.95
A1/16 80	S.B.; N.D.; AHSV‐6; HS 39/63; Kaalplaas, South Africa; 1963	30.49	14.43	87.87
A1/16 81	S.B.; N.D.; AHSV‐7; HS 31/62c; Kaalplaas, South Africa; 1962	33.18	15.75	87.88
A1/16 82	S.B.; N.D.; AHSV‐8; HS 10/62c; Kenya; 1962	30.25	13.29	87.87
A1/16 83	S.B.; N.D.; AHSV‐9; HS 90/61; Chad Fort Lamy; 1961	31.01	12.93	87.90
A1/16 84	Negative blood 1; –	Undet.	No *T*_P_	No *T*_a_
A1/16 85	S.B.; N.D.; AHSV‐2; Sen 2007; Diongo Village, Senegal; 2007	30.94	13.60	87.87
A1/16 86	S.B.; N.D.; AHSV‐4; Ken 2007; Kenya; 2007	30.45	13.08	87.95
A1/16 87	S.B.; N.D.; AHSV‐9; Ken 2006; Kenya; 2006	30.55	12.95	88.04
A1/16 88	S.B.; N.D.; AHSV‐2; Gha 2010; Polo Club, Ghana; 2010	35.04	15.63	88.04
A1/16 89	Negative blood 2; –	Undet.	No *T*_P_	No *T*_a_
A1/16 90	S.B.; N.D.; AHSV‐4; Eth 2010; Ethiopia; 2010	34.78	14.45	14.45
A1/16 91	S.B.; N.D.; AHSV‐6; Eth 2010; Ethiopia; 2010	32.09	12.97	87.94
A1/16 92	S.B.; N.D.; AHSV‐2; Eth 2010; Ethiopia; 2010	34.32	23.90	87.98^b^
A1/16 93	Negative blood 3; –	Undet.	No *T*_P_	No *T*_a_
A1/16 05	Negative blood 1 + 2; –	Undet.	No *T*_P_	No *T*_a_
A1/16 12	S.B.; –; Equine encephalosis virus (EEV) 10^‐2^	Undet.	No *T*_P_	No *T*_a_
RSArah1/01	S.B.; N.D.; AHSV‐1; (A501) OVI, South Africa; 1988	29.21	No *T*_P_	No *T*_a_
RSArah2/01	S.B.; N.D.; AHSV‐2; (OD) OVI, South Africa; 1988	22.43	14.02	88.30
RSArrah/03	S.B.; N.D.; AHSV‐3; (L) OVI, South Africa; 1988 and 1940	20.91	12.01	88.32
RSArrah/04	S.B.; N.D.; AHSV‐4; (Vryheid), OVI, South Africa; 1938	22.81	11.04	88.33
RSArrah/05	S.B.; N.D.; AHSV‐5; (VH), OVI, South Africa	21.72	13.00	88.23
RSArrah/06	S.B.; N.D.; AHSV‐6; (144), OVI, South Africa	20.12	10.08	88.10
KENrrah/07	S.B.; N.D.; AHSV‐7; (Karen), OVI, South Africa; 1952	23.59	12.55	88.26
RSArrah/08	S.B.; N.D.; AHSV‐8; (18/60), OVI, South Africa	24.08	12.44	88.14
PAKrrah/09	S.B.; N.D.; AHSV‐9; (7/60), OVI, South Africa	23.24	12.99	88.18
‐ve control		Undet.	No *T*_P_	No *T*_a_
BTV‐8	Bluetongue virus	28.00	No *T*_P_	No *T*_a_

a matrix; RT‐PCR result; serotype; strain; country of origin; year isolated.

b one *T*_a_ value obtained.

N.D, not determined, S.B, spiked blood.

–, +, ++, +++: original result obtained using RT‐PCR (Bremer et al., 1994).

Undet. *C*_T_ value undetermined by rRT‐PCR.

### RNA extraction

RNA was extracted in duplicate from 100 *μ*l of EDTA–blood using the KingFisher Flex‐automated extraction platform (ThermoFisher Scientific, Paisley, UK) and the LSI^™^ Magvet Universal isolation kit (ThermoFisher Scientific, UK). Viral RNA was eluted into 80 *μ*l of LSI elution buffer. The eluted RNA was stored at −20°C until analysis using rRT‐PCR was performed.

### Preparation of AHSV RNA in carrier RNA

To determine analytical sensitivity, a decimal dilution series (neat to 10^−10^) of RNA extracted from AHSV isolate (SENvvvv/09: an AHSV‐9 vaccine strain commonly used across Africa) was prepared in nuclease‐free water containing carrier RNA (1 *μ*g ml^−1^). RNA dilutions were tested in triplicate using the rRT‐PCR and RT‐LAMP assays.

### Real‐time reverse‐transcriptase PCR (rRT‐PCR)

rRT‐PCR was performed as described previously (Agüero et al., 2008) with minor modifications. Briefly, the fluorogenic rRT‐PCR was performed in a final volume of 25 *μ*l using a commercial kit (QuantiTect Reverse Transcription Kit; Qiagen, Manchester, UK). Duplicate 6 *μ*l aliquots of sample RNA was added to adjacent wells of a 96‐well optical reaction plate. RNA was denatured at 95°C for 5 min using a *Veriti* 96‐Well *Fast* Thermal Cycler (Applied Biosystems, Paisley, UK). Nineteen microlitres of one‐step reaction mix was prepared using 2X QuantiTect Probe RT‐PCR Master Mix (Qiagen) containing 1 ×  reaction mix: 1 *μ* m forward primer, 1 *μ* m reverse primer, 250 nm probe, 1.75 *μ*l molecular biology grade water and 0.2 *μ*l of enzyme mix were then added to each well. Primers and probe previously described by Agüero et al. (2008) were used. Amplification conditions consisted of reverse‐transcription (RT) performed at 48°C for 25 min followed by 95°C for 10 min and then 50 cycles of PCR, with 1 cycle consisting of 95°C for 15 s, 55°C for 35 s and 72°C for 30 s. rRT‐PCR was performed using an Applied Biosystems 7500 Fast real‐time PCR instrument (Applied Biosystems, UK).

### LAMP primer design

**Figure 1:**
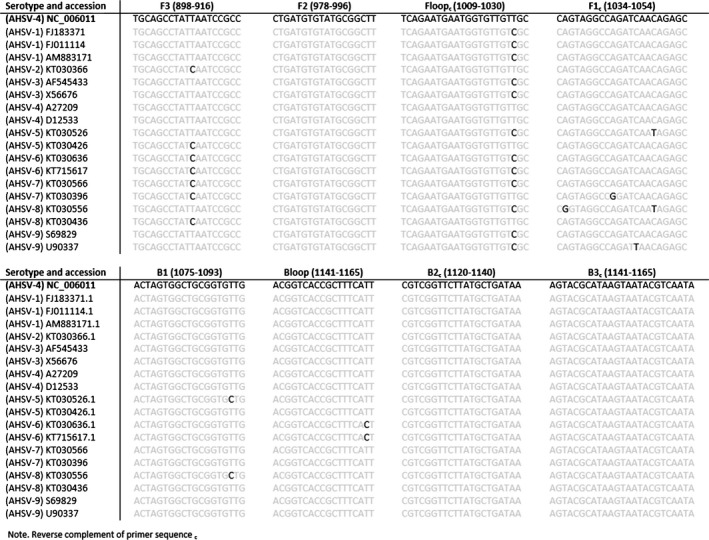
Alignment of RT‐LAMP assay primers against 19 African horse sickness virus published genomes.

Nineteen AHSV‐VP7 gene sequences (as encoded by segment 8) (Accession Numbers: NC_006011, FJ183371, FJ011114, AM883171, KT030366, AF545433, X56676, A27209, D12533, KT030526, KT030426, KT030636, KT715617, KT030566, KT030396, KT030556, KT030436, S69829, U90337), representing all nine serotypes, were obtained from GenBank. Sequences were aligned in Bioedit (version 7.0.5.3, Ibis Biosciences, Carlsbad, CA, USA) from which a highly conserved region was selected (spanning nucleotides 860‐1179: NC_006011) (Fig. 1). A single set of LAMP primers were designed from this region using LAMP Designer (OptiGene Ltd, Camberley, UK) (Table 2) using GenBank accession NC_006011.

**Table 2:** Oligonucleotide primers used for RT‐LAMP amplification of AHSV. Mapped to GenBank accession no.: NC_006011

Primer name	Length	Sequence 5′–3′	Position	Final concentration
F3	19‐mer	TGCAGCCTATTAATCCGCC	898–916	0.2 *μ* m (5 pmol)
B3	25‐mer	TATTGACGTATTACTTATGCGTACT	1141–1165	0.2 *μ* m (5 pmol)
FIP (F1c+F2)	40‐mer	GCTCTGTTGATCTGGCCTACTGCTGATGTGTATGCGGCTT	978–996; 1034–1054	0.8 *μ* m (20 pmol)
BIP (B1c+B2)	40‐mer	ACTAGTGGCTGCGGTGTTGTTATCAGCATAAGAACCGACG	1075–1093; 1120–1140	0.8 *μ* m (20 pmol)
Floop	22‐mer	GCAACAACACCATTCATTCTGA	1009–1030	0.4 *μ* m (10 pmol)
Bloop	18‐mer	ACGGTCACCGCTTTCATT	1095–1112	0.4 *μ* m (10 pmol)

### Reverse‐transcription loop‐mediated isothermal amplification (RT‐LAMP)

Extracted RNA was heated at 95°C for 5 min on a Veriti 96‐Well Fast Thermal Cycler (Applied Biosystems, UK). RT‐LAMP was performed in a total reaction mixture of 25 *μ*l containing: 15 *μ*l isothermal master mix ISO‐001 (OptiGene Ltd, UK), optimized primer concentrations (Table 3), 2 U AMV reverse transcriptase (New England Biolabs, Hitchin, UK), 5 *μ*l template and made up to volume with nuclease‐free water. RT‐LAMP reactions were run at 65°C for 30 min on a Stratagene Mx3005p (Agilent Technologies, Edinburgh, UK). All samples were tested in duplicate. ISO‐001 contains an intercalating dye, enabling results to be visualized using fluorescence collected at 1‐min intervals. A positive RT‐LAMP reaction was indicated by an exponential increase in fluorescence (δR) and the time to positivity (*T*_p_) defined when reactions reached a threshold increase of δR 1000.

**Table 3:** Effect of RT‐LAMP primer concentrations on performance of assay

Primer name	Reaction A		Reaction B		Reaction C		Reaction D	
	Concentration	Average *T*_P_	Concentration	Average *T*_P_	Concentration	Average *T*_P_	Concentration	Average *T*_P_
F3	0.2 *μ* m	8.70	0.2 *μ* m	9.95	0.2 *μ* m	9.19	0.2 *μ* m	9.98
B3	0.2 *μ* m		0.2 *μ* m		0.2 *μ* m		0.2 *μ* m	
FIP (F1c+F2)	2.0 *μ* m		1.6 *μ* m		1.2 *μ* m		0.8 *μ* m	
BIP (B1c+B2)	2.0 *μ* m		1.6 *μ* m		1.2 *μ* m		0.8 *μ* m	
Floop	1.0 *μ* m		0.8 *μ* m		0.6 *μ* m		0.4 *μ* m	
Bloop	1.0 *μ* m		0.8 *μ* m		0.6 *μ* m		0.4 *μ* m	

*T*_P_: Time to positivity.

To confirm that amplicons were AHSV‐specific, annealing analysis was performed on RT‐LAMP products using the Genie II (OptiGene Ltd, Camberley, UK), a portable fluorometer. LAMP products were heated to 98°C for 1 min and then cooled to 80°C decreasing at 0.05°C/s. Anneal temperature (*T*_a_) calculations were automated using Genie Explorer v0.2.1.1 software (OptiGene Ltd, Camberley, UK). Samples were considered positive if amplification had occurred and the LAMP product annealed in the AHSV amplicon‐specific temperature range of 87.0–88.6°C (mean *T*_a_ 87.8°C ± 0.8°C of 156 AHSV‐positive RT‐LAMP reactions).

### Statistical analysis

A receiver operator curve (ROC) was drawn using the statistical package R (R Core Team, 2014), using the pROC library (Robin et al., 2011a,b). For all curves generated, the rRT‐PCR was used as the gold standard.

## Results

### RT‐LAMP

Bioinformatics analysis showed that for all primer binding sites, there was a maximum of two mismatches (F3: 1, FIP [F2: 0, F1_:_ 2], Floop: 1, B3: 1, BIP [B1: 1, B2: 1], Bloop: 1) (Fig. 1). It was predicted that these mismatches would have minimal, if any effect, on assay performance because they are not present within the 3′ end of the primer binding site.

### RT‐LAMP optimization

To optimize the RT‐LAMP, four different primer concentrations were examined using RNA derived from (SENvvvv/09, AHSV‐9) (Table 3). *T*_P_ was similar across all primer dilutions; however, when RT‐LAMP was run for 60 min, a limited amount of late non‐specific amplification (confirmed by *T*_a_ analysis) was observed in reactions A‐C, which did not occur for reaction D (data not shown). Based on this analysis, reaction D (0.8 *μ* m FIPs/BIP; 0.4 *μ* m Floop/Bloop; 0.2 *μ* m F3/B3) was taken forward for evaluating samples from panels 1 and 2.

### Analytical sensitivity

**Figure 2:**
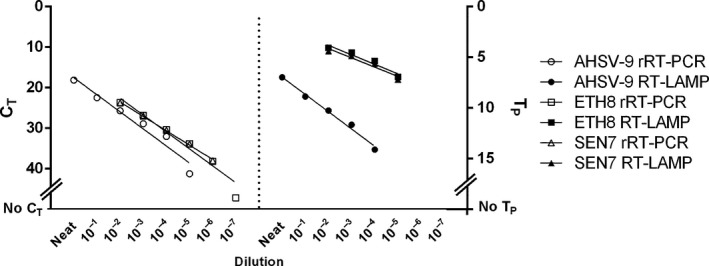
Comparative analytical sensitivity of African horse sickness virus RT‐LAMP and the gold standard rRT‐PCR.

A decimal dilution series of AHSV‐9 (SENvvvv/09) RNA and a dilution series of inactivated ETH8 and SEN7 spiked in blood were used to determine the analytical sensitivity of the RT‐LAMP compared to the rRT‐PCR (Fig. 2). The AHSV RT‐LAMP is comparable to the rRT‐PCR over a 4 log_10_ dilution range (*C*_T_ 20–36). The gold standard rRT‐PCR showed higher analytical sensitivity than the RT‐LAMP by one decimal dilution in all three dilution series (Fig. 2).

### Diagnostic sensitivity and specificity

**Figure 3:**
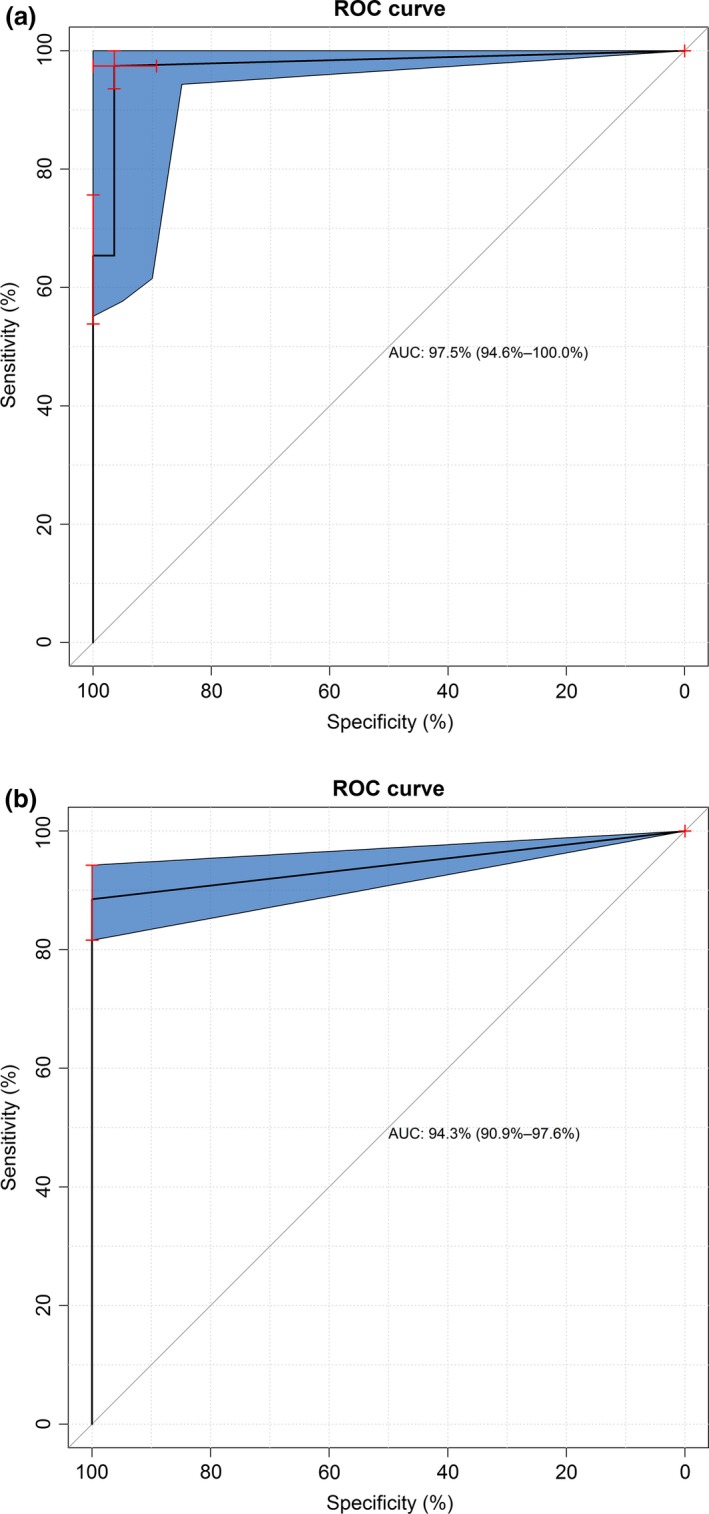
ROC curves comparing RT‐LAMP assay to the gold standard rRT‐PCR with either (a) no rRT‐PCR *C*_T_ cut‐off or (b) rRT‐PCR cut‐off *C*_T_ > 36.

A panel of 95 EDTA–blood samples comprising South African field samples, horse blood spiked with reference strains and related viruses (BTV, EEV), and another panel comprising nine AHSV reference laboratory strains were used to evaluate the sensitivity and specificity of the RT‐LAMP assay in comparison with the rRT‐PCR (Table 1; Fig. 3). There was complete concordance between rRT‐PCR and RT‐LAMP for 75/77 AHSV‐positive samples (with a *C*_T_ value <36) and 10/10 negative samples (with *C*_T_ undetermined), with the two samples negative by RT‐LAMP at the limits of analytical sensitivity (*C*_T_ = 34.92; *C*_T_ = 29.21). The diagnostic sensitivity of the assay was 90.3% when samples producing any *C*_T_ value were considered as positive. ROC curves were used to calculate the AUC (area under the curve) using rRT‐PCR as the gold standard: when no rRT‐PCR cut‐off was employed, the AUC was 0.943, whereas when a cut‐off (*C*_T_ > 36) was considered, the AUC was 0.975. There was no cross‐reaction of the RT‐LAMP assay with related viruses (EEV and BTV‐8) (Fig. 3).

## Discussion

AHS is a highly lethal disease of horses that causes a high number of deaths and economic losses in those African countries where the disease is endemic. But also, outbreaks of AHS have occurred twice in Europe in the past century, and BTV, which is closely related to AHS and is transmitted by the same route as AHS, has caused repeated outbreaks in the last two decades in several European countries, some of which have now become endemic (Herholz et al., 2008; Zientara and Sanchez‐Vizcaino, 2013). AHS therefore poses a significant threat to the global equine industry and remains the main barrier for international trade of horses between Africa and the rest of the world. Additional factors have also contributed to raise the global profile of AHS. Indeed, the expansion of equestrian sports in the Far East in recent years and the subsequent increase in the demand to facilitate the movement of high competition horses across international borders have led the OIE along with the International Federation of Horse Racing Authorities (IFHAA) and the Federation Equestre Internationale (FEI) to create the High Health High Performance horse (HHPH) concept. This scheme, currently under development, aims at defining the basic biosecurity conditions and standards under which high competition horses can be temporarily moved across international borders (Unt, 2014; Dominguez et al., 2016). The control of AHS plays a key role in the development of the HHP horse concept. Furthermore, since 2012, AHS became the only equine disease for which an official declaration of freedom is issued by the OIE.

Accurate, rapid, cost‐effective diagnostics are critical for the control of AHS outbreaks, for import/export testing of equines and surveillance. The availability of rRT‐PCR has improved the diagnosis of AHS, and these methods are now routinely used in AHS diagnostic laboratories. The value of rRT‐PCR has been recognized by the OIE, who sponsored an international ring trial aimed at harmonizing AHSV diagnostic methods used at the main AHSV diagnostic centres. This ring trial contributed to the validation of two tests which are now designated as OIE ‘recommended tests’ for the detection of AHSV (Agüero et al., 2008; Guthrie et al., 2013).

However, rRT‐PCR is exclusively performed on samples which have been carefully shipped to specialized laboratories, a process that can be time‐consuming. The availability of an RT‐LAMP assay for AHS, such as the one described in this study, would enable an aetiological diagnosis to be made within an hour of clinical or post‐mortem samples being collected. This capacity would enable the faster implementation of containment procedures within affected areas, which is of paramount importance for the control of outbreaks of AHS. Furthermore, rapid diagnosis in the field could lead to the immediate application of appropriate therapeutic interventions.

In this study, we describe the design of an RT‐LAMP assay for the detection of AHSV and the evaluation of its performance against i) clinical samples collected during recent outbreaks in South Africa, ii) historical isolates collected from other parts of Africa and iii) reference strains of AHSV. The diagnostic sensitivity of the RT‐LAMP assay is comparable to the recommended OIE rRT‐PCR assay. In our study, a number of field samples (originally tested as positive at origin using RT‐PCR) yielded negative results using the RT‐LAMP assay and also yielded high *C*_T_ values or a negative result when tested by the Agüero rRT‐PCR method. These results could be explained because the viral RNA in the original samples was diluted in negative horse blood and also because the RNA may have partially been degraded as a result of transport, freeze–thawing and manipulation in the laboratory. The analytical sensitivity of the RT‐LAMP assay was found to be one‐log lower than that of the rRT‐PCR assay (Fig. 2). However, the *C*_T_ values of the samples not detected by the RT‐LAMP assay were high, indicative of a low viral load.

Despite its slightly lower sensitivity, relative to the rRT‐PCR test used in this study, the AHSV RT‐LAMP could be used in its current format as a valuable complementary tool to standard, laboratory‐based rRT‐PCR assays. Samples from clinical cases of AHSV usually have high viral loads, which would fall within the detection range of the RT‐LAMP assay. This would allow for the rapid detection of positive samples and defined the RT‐LAMP assay as a valuable tool for use during outbreak investigations.

To facilitate the use of this assay in the field, future work should consider the removal of nucleic acid extraction steps, as described by Waters et al. (2014) and Howson et al. (2017); the lyophilization of the assay reagents, as shown by Howson et al. (2017); and the development of an alternative visualization strategy as described by Waters et al. (2014), Howson et al. (2017) and Fowler et al. (2016). It would be appropriate to extend the development of this RT‐LAMP assay to introduce the aforementioned modifications and testing the performance of the assay directly in the field during real outbreak investigations.

In conclusion, we have developed a novel, rapid AHSV RT‐LAMP assay with sufficiently high specificity and sensitivity that could be applied in the field and could improve early warning systems and control of AHS.
